# Management of SPN in France. Pathways for definitive diagnosis of solitary pulmonary nodule: a multicentre study in 18 French districts

**DOI:** 10.1186/1471-2407-8-93

**Published:** 2008-04-10

**Authors:** Kazem Alzahouri, Michel Velten, Patrick Arveux, Marie-Christine Woronoff-Lemsi, Damien Jolly, Francis Guillemin

**Affiliations:** 1 EA 4003, Nancy-University, Faculté de médecine, 9 avenue de la Forêt de Haye B.P. 184, 54500 Vandoeuvre lès Nancy, France; 2 Clinical Epidemiology Centre Inserm CIE6, Service d'Epidémiologie et Evaluation Cliniques, CHU de Nancy, 29 avenue du Maréchal de Lattre de Tassigny, 54000 Nancy, France; 3 Département d'Information Médicale, Centre Paul Strauss, Strasbourg, France; 4 Département d'Information Médicale, Centre Georges-François Leclerc, Dijon, France; 5 Service Pharmacie, Unité Evaluation Médico-économique, CHU de Besançon, France; 6 Département d'Informations Médicales, CHU de Reims, France

## Abstract

**Background:**

The process of diagnosis and management of solitary pulmonary nodules (SPNs) between 1 and 3 cm is not standardized. This multicentre study investigated how diagnosis of newly discovered SPNs is managed in routine practice.

**Methods:**

We examined 11,515 radiology reports of patients undergoing chest computed tomography (CT) at all 76 radiology centres in 18 French administrative districts covering 8,220,000 people. Information on diagnostic procedures and treatment administered from discovery to definitive diagnosis of SPN was collected prospectively.

**Results:**

We identified 152 cases of newly diagnosed SPNs. Follow-up was complete for 112 patients. The median number of diagnostic tests was 4 and the mean time to diagnosis was 41.4 days. Marked variability was observed in the sequence of diagnostic tests, and 8 diagnostic pathways were identified. Patients' characteristics and radiological features of SPNs influenced the number of tests performed. Referral by specialist, history of smoking and spiculated SPN predicted the performance of at least one invasive procedure (*P *< 0.01). Definitive diagnosis was a malignant disease in 30 patients (26%).

**Conclusion:**

The diagnosis of SPN is a complex process that physicians approach in markedly different ways. Implementing practice guidelines for managing the diagnosis of SPN requires clarification.

## Background

Solitary pulmonary nodule (SPN) is a common abnormality seen on radiology often detected incidentally by chest radiography or computed tomography (CT) [1,2]. Every year, American physicians investigate an estimated 150,000 patients with pulmonary nodules [3]. Because SPN is the initial radiographic finding in 10% to 20% of patients with lung cancer [4], the aim of evaluation and management is to promptly identify and bring to surgery all patients with operable malignant nodules, while avoiding unnecessary thoracotomy in those with benign nodules [5]. Concern about malignancy may lead physicians to adopt a surgical approach, but many radiographically detected lesions initially suspected to be cancerous are later proven not. The proportion of cases that turn out to be benign varied widely in published series [6]. Malignant disease is estimated to occur in 20% of patients with SPNs in the population and in 40% of those in surgical series [3,7,8].

The process of diagnosis may include CT scanning, whole-body positron emission tomography (PET), flexible bronchoscopy, transthoracic needle aspiration biopsy (TTNAB), transbronchic needle biopsy (Wang needle biopsy), video-assisted thoracoscopy, video-assisted thoracoscopic surgery, and thoracotomy. TTNAB is a more invasive way of obtaining a tissue diagnosis than bronchoscopy and Wang needle biopsy. It is much less invasive than surgery but also less reliable in ruling out malignancy. TTNAB and bronchoscopy are diagnostic; surgical resection has both diagnostic and therapeutic implications [9-11]. The choice of tests depends on many factors, including clinical features, results of relevant investigations, patient characteristics, and local care policy [7,12].

Guidelines published by the American College of Chest Physicians (ACCP) in 2003 address SPN evaluation [13], but National Comprehensive Cancer Network (NCCN) recommendations (v.2004) do not consider SPN as such. Some centres opt for early explorative surgery, whereas others carry out various imaging and invasive tests first. The literature reflects this lack of consensus [14,15].

Concerns regarding practice variations, quality of cancer care, and suboptimal patient outcomes seem to be well founded [16]. A high degree of variability might exist in the diagnostic process and management of SPNs [17]. Recent efforts have been made toward the implementation of evidence-based guidelines [18], but information about how physicians assess patients with SPNs in everyday practice is limited. We hypothesized that such variability in routine practice could be characterised by structured pathways and is affected by radiological features as well as other factors. The aim of the present study was to establish the diagnostic pathways that follow the discovery of SPN on chest CT to definitive diagnosis (malignant or benign) and to determine factors in decision making about SPN management.

## Methods

The present study was undertaken in an area of northeastern France covering 5 regions comprising a target of 18 health administrative districts with a population of about 8,220,000 people. The study was carried out under the auspices of the French Ministry of Health, and the study protocol was approved by the regional ethics committee and the national committee for confidential protection CNIL (**C**ommission **N**ationale de l'**I**nformatique et des **L**ibertés). As an observational study, informed consent was not required.

### Sampling subjects with SPN

Unfortunately, the definition of an SPN is controversial in the literature [13,19-21]. For the present study, we considered the radiologic definition described by a committee of the Fleischner Society on CT nomenclature: "coin lesion" or SPN defined as a "single round opacity, at least moderately well marginated and no greater than 3 cm in maximum diameter". An SPN less than 1 cm and ground glass nodules were not included in this SPN definition because recognizing this nodules depends greatly on available expertise and equipments [22].

A pilot study examining 268 consecutive sets of radiology reports of chest CT at 3 radiology centres revealed that data from these rapports supplemented with relevant information from the medical chart of the hospital or general practitioner [GP] were helpful in the study of SPN diagnosis. This pilot study also indicated that examining the results of chest CT performed for one week at each radiology centre would yield data about a sufficient of number of patients with SPNs in the whole area.

All the 76 radiology centres in the 18 health districts, community and teaching, public and private, used standard-dose chest CT. They all agreed to participate in this study over a randomly selected period of 6 consecutive weeks from May 2002 to March 2003. In the context of usual practice of each centre, all chests CT results were read by the radiologists of the centre. Radiologists used theirs locale standard criteria in reading chest CT and were informed by the patient's referral physician about the patient's clinical status and the results of any tests performed formerly. During the 6-week period, results of 11,515 chest CTs were read by radiologists. The radiology reports were sent to each patient's referring physician.

Five qualified clinical research assistants in charge of data collection were trained by a panel of the study investigators to ensure homogeneous results. The research assistants also included each newly diagnosed SPN recorded in the radiology reports. Cases were excluded if CT results showed evidence of metastasis or primary malignancy inside or outside the chest. All the included cases were verified by the panel of investigators. The GP and primary managing hospital physician were updated every 3 months on the process of diagnosis. Clinical research assistants collected data concerning age, sex, referring physician (generalist, specialist), appearance of SPN as recorded in the radiology reports (calcification within the nodule, appearance of spiculated nodule, mediastinal involvement or enlarged lymph nodes on CT), history of smoking (current or past smoker, never smoked), and dates and results of all investigations performed by the GP or any specialist physician (excluding laboratory and lung function tests). Diagnosis management was considered to be specialised if performed by a radiologist, chest physician, oncologist or thoracic surgeon.

For this study, we considered that the definitive diagnosis of SPN was established if there was histological evidence (malignant or benign) for the diagnosis. If the physicians involved in the patient care decided to discontinue the process of diagnosing without histological evidence, patients were followed up for 2 years after discontinuation of the diagnosis process. The 2-year prospective followup period was chosen because stability or no evidence of malignant disease for at least 2 years is a reliable indicator of a benign process [5,23], despite some investigators suggesting that even longer periods are necessary for confirmation [24].

### Statistical analysis

Procedures performed to establish the definitive diagnosis of SPN were analysed. The diagnostic pathway was defined as a set of tests used until definitive diagnosis. The duration of the process was calculated as the interval between the date of chest CT and that of any procedure after which physicians discontinued the diagnosis process of SPN. Quantitative variables are presented as median and ranges, categorical variables as proportions. Comparison of variables involved the Student's *t*-test or Mann-Whitney test for quantitative variables and chi-square or Fisher's exact tests for categorical variables.

A multivariate Poisson regression model suited to the distribution of number of tests was used to analyse the effect of the patients' characteristics and the radiological features of SPN on the number of tests performed before definitive diagnosis. A multivariate logistic regression model was used to examine the effect of the patients' characteristics and the radiological features of SPN on the probability of performance of at least one invasive test (surgical or non-surgical), adjusting for confounding variables. A multivariate Cox regression model was used to analyse the effect of the patients' characteristics and the radiological features of SPN on the time to definitive diagnosis. Two-tailed tests were applied with a significance threshold of 0.05. The Statistical Analysis System (version 8.2; SAS Institute; Cary, NC) was used for all analyses.

## Results

### Subject characteristics

Analysis of 11,515 radiology reports of chest CT over the 6 weeks identified 152 cases of newly diagnosed SPN. Patients were elderly (median 67.7 years; range 31.6–92.4, and predominantly male (72.4%); 122 (80.3%) were directly referred to radiology centres for chest CT by a specialist. Among 152 newly diagnosed SPN, 40 patients (24%) were lost to follow-up, including 6 patients who refused other investigations and 6 who died without evidence of definitive diagnosis. Baseline characteristics of the study population and patients with no follow-up data are in table 1. Patients without followup data were less likely to have a current or past history of smoking.

**Table 1:** Characteristics of patients with newly diagnosed SPN.

Variable	Patients with followup			Patients without followup			*p*
	
	Median (range)	N	%	Median (range)	n	%	
Age (yr)	67.7 (31.6–92.4)	112		70.4 (34.3–89.6)	40		0.44
Sex							
*Male*		82	73.2		28	70.0	0.70
*Female*		30	26.8		12	30.0	
Referral pattern							
*GP*		18	16.1		12	30.0	0.06
*Specialist*		94	83.9		28	70.0	
Smoking habits							
*Never smoked*		33	29.5		23	57.5	<0.01
*Current or past smoker*		79	70.5		17	42.5	
Nodule characteristics							
*Calcification within the nodule*		12	10.7		1	2.5	0.11
*Spiculated nodule*		32	28.6		16	40.0	0.18
*Mediastinal involvement*		31	27.7		13	32.5	0.56

GP = General Practitioner*

### Diagnostic procedures

For 57 patients, the benign diagnosis was based on imaging tests alone without histological evidence. For 39 of these, SPN was revealed on chest CT without further diagnostic procedures.

The median number of investigations was 4, and inconclusive test results were common. Factors with a potential influence on the number of tests performed are presented in table 2. In multivariate regression, the number of tests was significantly higher in patients older than 65 years (p = 0.02), referred by a specialist (p < 0.01), with current or past history of smoking (p < 0.01) and with spiculated nodules (p = 0.01).

**Table 2:** Number of tests performed prior to diagnosis according to patients and nodule characteristics (median and range).

Factor	n	No. tests prior to diagnosis	*p*^#^	*p*^##^
**Patient characteristics**				
Age(yr)				
*35–65*	52	3 (1–14)	0.43	0.02
*> 65*	60	4 (1–21)		
Sex				
*Male*	82	4 (1–21)	0.22	0.86
*Female*	30	3 (1–12)		
Referral pattern				
*GP*	18	2 (1–6)	<0.01	<0.01
*Specialist*	94	4 (1–21)		
History of smoking				
*Never smoked*	33	2 (1–12)	<0.01	<0.01
*Current or past smoker*	79	5 (1–21)		
**Nodule characteristics**				
Calcification within the nodule				
*Yes*	12	2 (1–14)	0.04	0.76
*No*	100	4 (1–21)		
Spiculated nodule				
*Yes*	32	5 (1–14)	0.02	0.01
*No*	80	3 (1–21)		
Mediastinal involvement				
*Yes*	31	4 (1–21)	0.4	0.19
*No*	81	4 (1–12)		

GP = General Practitioner.

*p*^# ^In Mann-Whitney test.

*p*^## ^In multivariate Poisson regression model.

Apart from CT of the chest, imaging tests carried out were (in decreasing order of frequency) 31 CT of complete abdomen (27.6%); 19 CT or magnetic resonance imaging of the brain (16.9%); 18 ultrasonography of abdomen (16%); 10 bone scan (8.9%); 5 CT of cervix (4.4%) and 2 whole-body positron emission tomography (1.7%). In 25 cases, an imaging test was repeated at least once before a definitive diagnosis was reached.

Fifty-five patients underwent invasive diagnostic techniques to evaluate SPN 48 flexible bronchoscopy (42.8%), 9 transthoracic needle aspiration biopsy (8%), 3 transbronchic (Wang) needle biopsy (2.7%) and 10 exploratory thoracotomy (8.9%). In 15 cases, an invasive test was repeated at least once before a definitive diagnosis was reached.

### Diagnostic pathways

**Figure 1:**
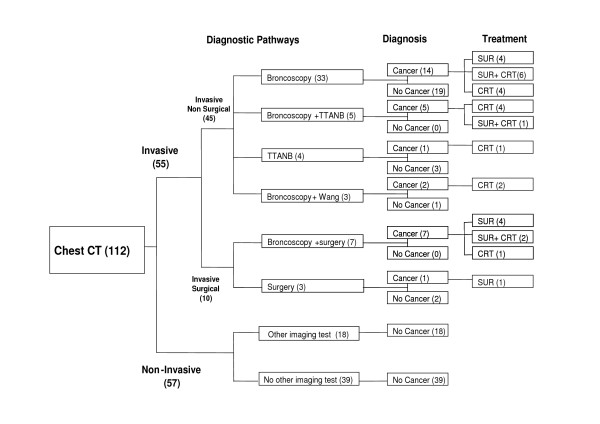
**Diagnostic pathways prior to definitive diagnosis of SPN**. Numbers in parentheses are numbers of patients. (SPN = solitary pulmonary nodule; CT: computed tomography; TTNAB: trans-thoracic needle aspiration biopsy; Wang: Wang needle biopsy; SUR: surgery; CRT: chemoradiotherapy).

A high level of variability was observed in the sequence of tests performed. Eight diagnostic pathways were identified. They fell into 3 categories: pathways based on imaging alone, with no invasive investigations (noninvasive); those based on at least 1 surgical procedure (invasive surgical); and those based on at least 1 invasive nonsurgical procedure (invasive nonsurgical) (Fig. 1). For 57 patients, the process of diagnosis was non-invasive and for 55 invasive (surgical and nonsurgical). In 33 cases, the definitive diagnosis was made from bronchoscopy after chest CT. Chest CT followed by TTNAB was sufficient to establish the definitive diagnosis in 4 patients.

### Determinants of diagnostic pathways

Table 3 shows the univariate and multivariate odds ratios (ORs) of variables related to the patient characteristics and the radiological features of SPN predicting the use of at least one invasive test (surgical or nonsurgical). In the univariate analysis, male sex, current or past history of smoking, referral from a specialist physician, spiculated nodule, and mediastinal involvement all increased the likelihood of at least one invasive test conducted. Multivariate analysis showed that patients referred by a specialist physician (OR = 6.6) with current or past history of smoking (OR = 32.9) and a spiculated nodule (OR = 5.7) were significantly more likely to undergo at least one invasive test (p < 0.01). A total of 30 patients (26.8%) were given a diagnosis of cancer.

**Table 3:** Factors influencing whether patients with SPN undergo at least one invasive test (Odds ratio [OR] and 95% confidence interval [CI]).

Factor	n	Unadjusted OR of receiving invasive test (CI)*	Adjusted OR of receiving invasive test (CI)^+^	*P*^*$*^
**Patient characteristics**				
Age				
*35–65*	52	0.8 (0.3–1.7)	1.5 (0.5–4.3)	0.38
*> 65*	60	1	1	
Sex				
*Male*	82	2.9 (1.2–7.2)	0.5 (0.1–3.8)	0.57
*Female*	30	1	1	
Referral pattern				
*Specialist*	94	4.1 (1.2–13.5)	6.6 (1.4–30.3)	0.01
*GP*	18	1	1	
History of smoking				
*Current or past smoker*	79	19.2 (5.3–68.8)	32.9 (4.3–247.7)	<0.01
*Never smoked*	33	1	1	
**Nodule characteristics**				
Calcification within the nodule				
*Yes*	12	0.1 (0.1–0.8)	0.5 (0.1–4.2)	0.54
*No*	100	1	1	
Spiculated nodule				
*Yes*	32	4.7 (1.8–11.8)	5.7 (1.6–19.5)	<0.01
*No*	80	1	1	
Mediastinal involvement				
*Yes*	31	1.9 (0.8–4.6)	1.4 (0.4–4.3)	0.49
*No*	81	1	1	

GP = general practitioner

*In univariate logistic regression models

+In multivariate logistic regression model

^$^p values for the factors are the Wald statistics for the estimates in the model

### Duration of process of diagnosis

Time to definitive diagnosis was 41.4 days, on average, and more than 30 days in 40% of cases. Among patients exhibiting calcification within the lesion, the median time to diagnosis was 26.5 days. Mediastinal involvement was reported in 31 patients, among whom the median interval between the chest CT and definitive diagnosis was 25 days (Table 4). Cox multivariate regression analysis showed no effect of the radiological appearance of the nodule or patients' characteristics on time to definitive diagnosis.

**Table 4:** Time between first test and diagnosis according to nodule characteristics. (Median and interquartile).

Factor	n	Days to diagnosis	*p*^#^	*p*^##^
**Patient characteristics**				
Age				
*35–65*	52	24 (4–61.5)	0.5	0.33
*> 65*	60	28 (11.5–53.5)		
Sex				
*Male*	82	26 (8–63)	0.4	0.42
*Female*	30	25.5 (0–46)		
Referral pattern				
*GP*	18	18 (0–33)	0.10	0.07
*Specialist*	94	28 (8–62)		
History of smoking				
*Never smoked*	33	13 (0–35)	<0.01	0.17
*Current or past smoker*	79	29 (11–63)		
**Nodule characteristics**				
Calcification within the nodule				
*Yes*	12	26.5 (5–51)	0.4	0.34
*No*	100	25.5 (0–25.5)		
Spiculated nodule				
*Yes*	32	33 (25.5–65.5)	0.02	0.41
*No*	80	14.5 (3.5–56.5)		
Mediastinal involvement				
*Yes*	31	25 (11–62)	0.29	0.74
*No*	81	27 (6–61)		

GP = General Practitioner

*p*^# ^In Mann-Whitney test.

*p*^## ^In multivariate Cox regression model.

### Patient outcome

Among 112 patients with complete follow-up data, the definitive diagnosis was a malignant disease in 30 patients (26, 78%). Among 31 patients with mediastinal involvement at the first observation of SPN, 16 (51, 61%) cancer was diagnosed at the end of process of diagnosis. The mediastinal involvement was more likely to be associated with cancer (p = 0.005).

Table 5 shows the histological classification in the 30 patients with a definitive diagnosis of cancer as follows: squamous cell carcinoma (13; 43.3%), adenocarcinoma (10; 33.3%), Bronchioloalveolar carcinoma (2; 6.7%), large cell (2; 6.7%) and Small cell lung carcinoma (1; 3.3%), SPN was the first manifestation of primary colon cancer in one patient and of lymphoma in another. Information regarding stage was available in 26 patients (86.6%). The details of the stage of lung cancer at presentation are shown in table 6. Lobectomy with intent to cure was performed in 15 patients; lobectomy was also the diagnostic test in 8 of these patients. Eight patients received chemotherapy or radiotherapy in addition to surgery.

**Table 5:** The histological classification in the 30 patients with a definitive diagnosis of cancer.

Histological type	n	%
Squamous cell carcinoma	13	43.3
Adenocarcinoma	10	33.3
Bronchioloalveolar carcinoma	2	6.7
Large cell carcinoma	2	6.7
Small cell lung carcinoma	1	3.3
Lymphoma	1	3.3
Unspecified lung cancer	1	3.3

**Table 6:** The stage of disease in the 30 patients with a definitive diagnosis of cancer.

Stage of cancer	n	%
IA	6	20
IB	8	26.7
IIA	1	3.3
IIB	3	10
IIIA	2	6.7
IIIB	3	10
IV	3	10
Non reported in medical report	4	13.3

## Discussion

The present paper describes the diagnostic process in a representative sample of patients with newly diagnosed SPN identified from 11,515 radiology reports of chest CT at all radiology centres in northeastern France. Reflecting physicians' usual practices, the process of diagnosing new SPN varied widely over 8 diagnostic pathways and was often protracted, with many patients not undergoing histological examination of the SPN. The radiological features of SPN influenced the number of tests performed prior to definitive diagnosis and the likelihood of performing at least one invasive test. Although physicians attempted to minimise the delay until definitive diagnosis in many cases, a considerable subset of patients underwent a prolonged period of diagnosis and inconclusive tests were common.

The evaluation of a solitary pulmonary nodule is complex. Management decisions are based on clinical history, size and appearance of the nodule, and feasibility of obtaining a tissue diagnosis, so some variability in management may be justified as many considerations must be taken into account in diagnosing SPN. Abnormality of any form seen on chest CT is a worry for the patient during the entire process. So differences in patient's preference for imaging or an invasive test might affect physician decision making. In a large population, as in this study of 8 million people potentially referable for chest CT, local policy of the centres and education of physicians involved in the management of SPN can account for physicians' differing choices for tests and the high number of tests performed. Since a criterion standard pathway does not exist and guidelines do not recommend a "good practice" sequence of diagnostic tests, investigations ordered for SPN diagnosis might reflect the physician's own awareness of the options and their cost. Reasons for variations observed in practices can be grouped into three categories: those related to professional uncertainty, to external constraints, and to diffusion of new knowledge and practices. Combinations of clinical factors such as the characteristics of nodules, and structural ones such as the availability of resources, lead to different local standards of medical care. The present findings are in accordance with previous studies describing high variation in physician practice patterns in other settings [25].

To date, no published data exists on the diagnostic pathways for SPN. Our study differs in many ways from the few others that have examined the diagnostic management of SPN. First, our study is based on information from randomly selected radiology reports representative of a large population in 18 health districts and therefore reflects usual practice in France and is not biased by the selection of cases. Clearly, only patients with nodules with suggestive characteristics seen on imaging are referred to a specialised centre to undergo surgery for characterization, and, thus, studies based on such data have a marked selection bias. Second, our search included the results of all chest CT. Thus, many of the SPNs were incidental findings on chest CT. In the radiology literature, studies of SPN have been developed in the context of lung cancer screening programs [20,21,26]. However, subjects who undergo lung cancer screening in most countries are selected on the basis of age, substantial smoking history, absence of serious co-morbid disease, and willingness to participate in all necessary follow-up imaging and intervention. Moreover, these programs tend to have a standardized protocol for follow-up of subjects and for early cure. Therefore, subjects whose nodules are detectedincidentally during chest CT performed for other reasons should not necessarily be managed in the same way as subjects in a screening program.

The literature reports important variations in the definition of the time to definitive diagnosis of subjects with suspected lung cancer. Different intervals were used, from the first symptoms until the treatment [27,28]. These endpoints reflect the different concerns of investigators. These clinical endpoints are all relevant but not comparable. In regard to the diagnosis of lung cancer, our data suggest that the interval between the initial CT and definitive diagnosis is likely long. The need to shorten the interval to diagnosis is likely supported by several studies [29-32]. Extended intervals between symptoms, initial chest CT, diagnosis and surgery may give the primary tumour the time to double in size and may increase the likelihood of local and distant spread.

Although this investigation provides new information about managing the diagnosis of newly discovered SPN in routine practice, several limitations should be addressed. First, the study protocol defined nodule as round opacity, with a maximum diameter of 3 cm and a minimum diameter of 1 cm. While this provides an idealized definition of a lung nodule demonstrated on CT scans, the natural complexities of biologic systems make the practical application of such a definition difficult [33]. We used the more possible generic term to describe a solitary pulmonary nodule and this study was designed to assess all types of nodules rather than one specific type as the first question posed by our study investigated how SPN is managed in daily practice. Our hypothesis was that nodule characteristics and mediastinal lymph node enlargement could influence clinician strategies to evaluate patients with pulmonary nodule so we included the information about mediastinal involvement in our analysis. We chose to exclude small nodules because recognizing nodules less than 1 cm depends greatly on available expertise and equipment [19]. However, the clinical importance of these small nodules differs from that of larger nodules. This issue has been highlighted in recent publications on CT screening for lung cancer, and the positive relationship of lesion size to likelihood of malignancy has been clearly demonstrated. Second, it was difficult to obtain detailed follow-up information on some patients. Complete records of ambulatory and hospital diagnostic procedures were not available in 40 cases. Given that it was not possible to ascertain diagnosis at the moment of the first detection of SPN on chest CT by the radiologists these patients were considered as lost to follow-up. Compared with the study population, members of this group were less likely to be current or past smokers. The lack of a centralised patient-based information system in France led to a relatively high proportion of cases with unavailable data. As likelihood of cancer is low in patients without history of smoking we could imply that physician have less attention to require another assessment (invasive or non invasive) for these patients. Third, some prognostic factors, for example, a history of asbestos exposure or co-morbid condition of patients were not taken into account. Differences in prognosis may have accounted for the physicians' differing choices for tests. Finally, for 112 patients, several problems arose concerning the order in which tests were conducted. The many possibilities resulted in a number of combinations, with some diagnostic pathways being used in only 3 or 4 patients.

With increasing clinical governance in the health system in France, emphasis is being placed on identifying the best practice and avoiding delays in diagnosis of lung cancer that are potentially detrimental to the patient[5,23], Lack of a clear protocol for diagnosis warns of potential for failure to recognize primary malignant disease. The present study looked at the pathways of care from the point of referral by a radiologist recognizing the presence of SPN to definitive diagnosis. The findings can therefore be used to inform health service planning about real practice, to identify areas for improvement, and to update guidelines for managing diagnosis of SPN. Future practice guidelines for SPN diagnosis should be supported by more evidence and should take into account the features of SPN revealed by chest CT and the patient's clinical characteristics. Clarifications would derive more attention as initiation of effective treatment may improve survival.

PET imaging can reveal foci of lung cancer not seen using other imaging techniques It reported a sensitivity of 96.8% and a specificity of 77.8% for any focal lung lesion; and figures of 93.9% and 85.8%, respectively for pulmonary nodules [34,35]. PET was not available in France at the time when this study was conducted. However some bordering patients could be referred by the generals practitioners, if need, to perform PET in a nearest foreign country. Recently, many French hospitals have decided to adopt it. This study provides baseline information to measure improvements in the care of patients with SPN after the implementation of PET imagining. Its role in daily clinical practice as a means of simplifying and improving the final selection of patients for surgery is currently under study.

## Conclusion

In conclusion, our data suggest that the interval between the initial CT and the definitive diagnosis of SPN is likely long in northeastern France and efforts should be made to shorten this interval. Specific information could be provided about which patients follow each diagnostic pathway, but given the observational design of this study, more work is necessary to better define a "minimally invasive" optimal pathway in the definitive diagnosis of SPN.

## Competing interests

The author(s) declare that they have no competing interests.

## Authors' contributions

KA, participated in the design of the study, acquisition analysis and interpretation of data. MV, PA, MCWL, DJ and FG conceived of the study, participated in its design and coordination, involved in drafting or revising the manuscript critically and helped to draft the manuscript. All authors read and approved the final manuscript.

## Pre-publication history

The pre-publication history for this paper can be accessed here:


